# Stewards for future: Piloting a medical undergraduate elective on antimicrobial stewardship

**DOI:** 10.3205/zma001733

**Published:** 2025-02-17

**Authors:** Cihan Papan, Barbara C. Gärtner, Arne Simon, Rachel Müller, Martin R. Fischer, Dogus Darici, Sören L. Becker, Katharina Last, Stefan Bushuven

**Affiliations:** 1 Saarland University, Institute of Medical Microbiology and Hygiene, Center for Infectious Diseases, Homburg, Germany; 2 University Hospital Bonn, Institute for Hygiene and Public Health, Bonn, Germany; 3 Saarland University, Department of Pediatric Hematology and Oncology, Homburg, Germany; 4 Saarland University Medical Center, Pharmacy, Homburg, Germany; 5 LMU University Hospital, LMU Munich, Institute of Medical Education, Munich, Germany; 6 University of Münster, Institute of Anatomy and Molecular Neurobiology, Münster, Germany; 7 Health Care Association District of Constance, Konstanz, Germany; 8 University of Freiburg, Faculty of Medicine, Department of Anesthesiology and Critical Care, Freiburg/Brsg., Germany

**Keywords:** antimicrobial resistance, antimicrobial stewardship, antibiotic stewardship, medical undergraduate education, case-based learning, problem-based learning

## Abstract

**Background:**

The rise of antimicrobial resistance as leading infection-related cause of death will necessitate trans-sectoral efforts on a global level. While many antimicrobial stewardship (AMS) incentives target healthcare workers, addressing undergraduates offers new and hitherto neglected opportunities.

**Methods:**

We describe the pilot phase of a novel undergraduate elective (“stewards for future”, SFF) for medical students at the Saarland University, Germany, between 2021 and 2023. We focused on knowledge and attitudes relevant to AMS. To allow for full immersion, we applied case-based learning, problem-based learning, and peer teaching in a small group teaching format spanning 15 hours, including AMS ward rounds. We obtained students’ pre- and post-course self-assessment regarding AMS topics using 5-point Likert scales modified from the previously published ASSURE elective, as well as their subjective experience using the German short intrinsic motivation inventory.

**Results:**

Over four terms, 23 undergraduate medical students from the clinical phase participated in the elective. Participants reported an increase in their ability to explain the concept of AMS (mean and standard deviation, pre 3.26±0.94 vs. post 4.74±0.44, p<0.0001), their confidence in choosing the appropriate antibiotic (pre 2.22±0.78 vs. post 3.57±0.58, p<0.0001), their ability to judge potential drug side effects (pre 2.09±0.72 vs. post 3.43±0.71, p<0.0001), their confidence in communicating with colleagues about antibiotics (pre 2.30±0.86 vs. post 3.52±0.83, p<0.0001), their understanding of diagnostics as an AMS tool (pre 4.22±0.41 vs. post 4.91±0.28, p<0.0001), and their ability to evaluate the roles of all AMS team members including their own (pre 2.52±0.77 vs. post 4.13±0.68, p<0.0001). Participants reported having enjoyed the course (4.6±0.5), while they were moderately satisfied with their performance (3.8±1.0). Pressure and anxiety levels were reported to be low (1.8±0.9 and 2.0±1.0 each).

**Conclusions:**

Student participants of the elective SFF reported increased competencies relevant to AMS, while enjoying the course format. Sustainability and scalability will ultimately depend on the implementation into the core curriculum.

## Zusammenfassung

**Hintergrund:**

Antibiotikaresistenzen und damit einhergehende Infektionen sind mit einer sehr hohen Krankheits- und Sterblichkeitslast assoziiert und erfordern einen sektorenübergreifenden, global ansetzenden Umgang. Während in den letzten Jahren zahlreiche Antibiotic Stewardship (ABS) Maßnahmen, beispielsweise als Kurse für Ärzt:innen, initiiert wurden, sind Studierende bislang als Adressaten unterrepräsentiert.

**Methodik:**

In diesem Pilotprojekt wird die Konzeption und Einführung eines neuen Wahlpflichtfaches („Stewards for Future“, SFF) für Studierende an der Medizinischen Fakultät der Universität des Saarlandes im Zeitraum 2021 bis 2023 beschrieben. Der Fokus des Lehrformates lag auf der Vermittlung ABS-relevanter Kompetenzen, bezogen auf Wissen und Haltung. Die dabei eingesetzten Lehrmethoden waren Fall-basiertes und Problem-orientiertes Lernen, Peer Teaching in Kleingruppen, sowie Visiten am Patientenbett. Das Wahlpflichtfach umfasste 15 Stunden. Zu Beginn und nach Abschluss des Wahlpflichtfaches erfolgte eine ABS-Themen bezogene Selbsteinschätzung mittels 5-Punkt-Likert-Skalen auf der Basis eines zuvor publizierten Fragebogens. Zudem wurde nach Abschluss das subjektive Erleben anhand des deutschsprachigen Kurzfragbogens zur intrinsischen Motivation erhoben.

**Ergebnisse:**

Über einen Zeitraum von vier Semestern nahmen insgesamt 23 Medizinstudierende des klinischen Abschnitts am Wahlpflichtfach teil. In der Selbsteinschätzung durch die Teilnehmenden konnte eine Zunahme von zu Beginn (vorher) bis nach Abschluss (nachher) des Wahlfachs für folgende Angaben festgestellt werden: ihre Fähigkeit, die Grundlagen und Bedeutung von ABS zu erklären (Mittelwert und Standardabweichung vorher 3,26±0,94 vs. nachher 4,74±0,44, p<0,0001); die Sicherheit in der Auswahl von Antibiotika (vorher 2,22±0,78 vs. nachher 3,57±0,58, p<0,0001); ihre Fähigkeit, das Nebenwirkungspotential von spezifischen Antibiotika zu bewerten und in ihre Auswahl einfließen zu lassen (vorher 2,09±0,72 vs. nachher 3,43±0,71, p<0,0001), ihre Sicherheit in der Kommunikation mit Kolleg:innen bezüglich Antibiotika (vorher 2,30±0,86 vs. nachher 3,52±0,83, p<0,0001), das Verständnis der Bedeutung der Infektionsdiagnostik für den rationalen Einsatz von Antibiotika (vorher 4,22±0,41 vs. nachher 4,91±0,28, p<0,0001), und die Fähigkeit, ihre Rolle als Steward im interprofessionellen Team einzuordnen und die Rollen der Anderen im Team zu kennen (vorher 2,52±0,77 vs. nachher 4,13±0,68, p<0,0001). Die Teilnehmenden gaben an, dass ihnen der Kurs Spaß gemacht hätte (4,6±0,5), wobei sie moderat zufrieden mit ihrer eigenen Leistung waren (3,8±1,0). Das Gefühl unter Druck oder angespannt zu sein, wurden als niedrig eingeschätzt (1,8±0,9 und 2,0±1,0).

**Schlussfolgerung:**

Die Teilnehmenden des Wahlpflichtfachs SFF berichteten von angestiegenen Kompetenzen, die für ABS relevant sind, und hatten zugleich Spaß am Lehrformat. Die Nachhaltigkeit und Skalierbarkeit des Projektes werden letztlich von der Implementation in das Curriculum abhängen.

## Introduction

In the past decades, antimicrobial resistance (AMR) has continued to grow into one of the biggest threats to human health. As of 2019, at least 1.27 million deaths were attributable to infections with resistant pathogens, thereby rendering AMR the leading infectious diseases cause of mortality worldwide [1]. One main driver of surging AMR is the injudicious overuse of antibiotics across all healthcare sectors.

Despite the increasing topical importance, medical students feel insufficiently prepared to tackle this challenge [2]. Previous surveys among medical undergraduate students have demonstrated that they not only acknowledge the interplay between antibiotic overuse and AMR; they also regard antibiotic overuse as unethical [3], and also wish to have more targeted teaching pertaining to the rational use of antibiotics [4], [5]. Another aspect pertains to the higher psychosocial burden in healthcare workers when dealing with patients with multidrug-resistant organisms [6], [7], which further contributes to the relevance of AMR in education. The rational use of antibiotics, also coined antimicrobial stewardship, has been implemented in many clinical settings and countries, with courses mainly targeting postgraduate, specialist-level physicians working in secondary or tertiary care [8]. However, antimicrobial stewardship (AMS) also entails behavioral change, which becomes challenging with the healthcare professionals’ increasing age and experience, necessitating highly sophisticated interventional strategies, often only to reach miniscule effects [9], [10]. Previous studies have also demonstrated that older physicians are more prone to prescribe antibiotics than their younger peers [11], [12]. In addition, most antibiotics are prescribed in the primary care sector [13], which has been a neglected area with regard to AMS activities thus far, compared with the focus on inpatients. Given the demand and need of medical students mentioned above, it appears reasonable and desirable to consider possibilities of undergraduate-level implementation, to make use of imprinting or behavior shaping at an early stage [14], while addressing students’ knowledge and skills, but also their attitude. Given the fact that a substantial percentage of students will ultimately work in primary care [15], and the reported need among primary care physicians for AMS-related education [16], we conceived and piloted an elective for medical undergraduate students at Saarland University. 

## Project description

This is a retrospective analysis of an elective course that we conceptualized for medical undergraduate students in the clinical phase (“Stewards for Future – Training of Medical Students to Combat Antimicrobial Resistance”) at Saarland University, Germany. The conception of this elective followed the principles and concept of curriculum development laid out by Kern [17]. 

### Step 1: Problem identification and general needs assessment

Based on the problem identification and needs assessment described in the introduction, the main focuses were knowledge and attitudes relevant to AMS.

### Step 2: Needs assessment of targeted learners

Internal review of students’ performance in microbiology and infectious diseases exams and of the local curriculum, as well as informal interviews with doctoral candidates in medical microbiology and other students confirmed the general needs assessment for the Saarland University.

### Step 3: Goals and objectives

The main objective of the elective was based on the NKLM Version 2.0 (Nationaler Kompetenzbasierter Lernzielkatalog Medizin; in English: national competency-based catalogue of learning objectives medicine), specifically, on the objective VII.3-19.1 which addresses the basic concept of antimicrobial stewardship ([https://nklm.de/zend/objective/view/id/10006975/essential/yes/lve/212157]; in English: The graduate masters the basics of anti-infective therapy. They can explain the rational use of antibiotics to avoid the development of resistance and take into account the corresponding principles in their own actions. […] Students are able to initiate diagnostics and antimicrobial therapies while considering the rational use of antimicrobials by means of critical indication assessment, critically review of existing antimicrobial therapies and adapt them if necessary.). This specific learning objective was complemented in part by other, syndrome-specific objectives touching upon AMS (e.g., V.01.1.1.102 for acute otitis media, V.01.1.1.60 for tonsillopharyngitis, VI.04-01.2.5 for pneumonia, VI.05-01.7.4 for catheter-associated infections, VI.05-01.8.10 for sepsis, V.01.1.1.96 for meningitis, VI.01-01.11.8 for endocarditis).

### Step 4: Educational strategies

We applied case-based learning, problem-based learning, and peer teaching in a small group teaching format to allow for contextual, collaborative, constructive and self-paced learning [18]. Cases were derived from real life clinical work and/or from CASUS, an e-Learning platform with virtual patients accessible for healthcare workers involved in teaching at German medical faculties [https://lmu.casus.net/pmw2/app/homepage.html]. Each session was introduced with a short (20 minutes) lecture. The topics covered in the lectures were

1. AMS basics and principles;
2. development of an antibiotic prescription framework; antibiotics 101:
3. spectrum, dosing and dose adjustment, and bioavailability;
4. guidelines-based management of the most frequent infectious diseases;
5. managing patient behaviour through communication;
6. the role of diagnostics as an AMS tool;
7. the interplay between infection prevention and control and AMS.

These topics were chosen because they cover the fundamental principles of antimicrobial treatment and antimicrobial stewardship.

### Step 5: Implementation

The blueprint of the elective was developed throughout the summer term 2021. During this preparatory phase, administrative and political support was obtained, including alignment with the Dean’s office. The elective was first offered in the winter term 2021/2022, and repeated through the summer term 2023, for a maximum of 25 participants per semester. The elective consisted of 15 hours in total, distributed over seven sessions (2 hours each), in addition to one AMS ward round in the university hospital.

### Step 6: Evaluation and feedback

We obtained students’ pre- and post-course self-assessment regarding AMS topics using 5-point Likert scales (ranging from 1=strongly disagree, to 5=strongly agree). To this end, we modified the previously published set of items from Wang and colleagues [19]. The survey comprised the following statements for which the participants were asked to state their agreement using the 5-point Likert scale:

- I perceive AMS as an important topic;
- I am able to explain the concept of AMS;
- I am confident in choosing the appropriate antibiotic;
- I am able to judge potential antibiotic side effects;
- I am confident in communicating with colleagues about antibiotics;
- I have an understanding of diagnostics as an AMS tool;
- I am able to evaluate the roles of AMS team members (including my own).

In addition, we assessed their intrinsic motivation using a German, short version of the intrinsic motivation inventory by Deci and Ryan translated and validated by Wilde and colleagues [20], [21], which includes subscales for interest and enjoyment; perceived competence; pressure and tension; and perceived choice (see table 1 (Tab. 1)).

### Statistics and ethics

All data were obtained anonymously. To allow for pre- and post-course comparison by matching intraindividual data, participants used a non-identifiable code. The study was exempt by the Ethics committee at the Chamber of Physicians of Saarland (Ärztekammer des Saarlandes).

We performed paired Wilcoxon rank sum tests to compare pre- and post-course self-assessment scores. The statistical significance level was set at 0.05. We used R (Version 4.3.1) for data analysis and visualization, including the packages “ggplot2”, “likert”, and “wesanderson” [22], [23], [24]. 

## Results

Over four terms, a total of 23 students participated in the elective, of whom 18 were women (78%). The number of students per course was six in all but the summer term 2022, in which five participated. The mean age of participants was 24.4 years (±3.0 standard deviation, SD), and their median medicine-specific semester was 7 (out of a total of 12 semester; interquartile range, IQR, 6-9). 

**Figure 1:**
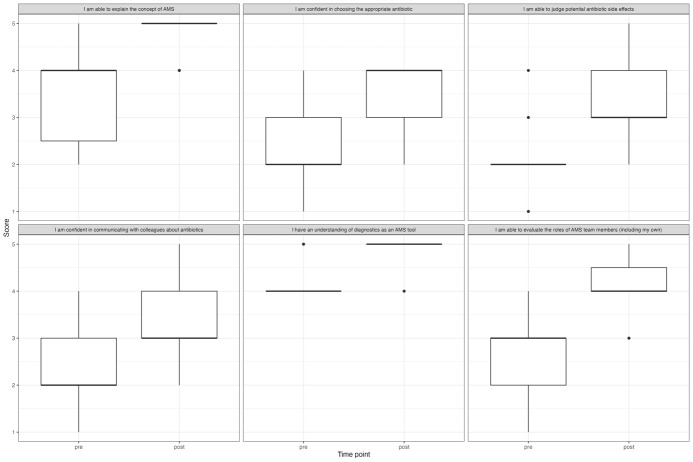
Comparison of pre- and post-course self-assessment for different antimicrobial stewardship related domains, aggregated for all 23 participants

We observed an aggregated increase across all items of the self-assessment survey (see figure 1 (Fig. 1)), with the highest mean score increase for the item “evaluate roles of AMS team members”, which increased from 2.52 (±0.77) to 4.13 (±0.68) (mean Δ1.6; *P*<0.0001), followed by “ability to explain the concept of AMS” (3.26 vs. 4.74, mean Δ1.48; *P*=0.0001), “confidence in choosing the appropriate antibiotic” (2.22 vs. 3.57; *P*=0.0001) and “ability to judge potential drug side effects” (2.09 vs. 3.43; *P*<0.0001) (both mean Δ1.35), “confidence in communicating with colleagues about antibiotics” (2.30 vs. 3.52, mean Δ1.22; *P*=0.0003), “understanding of diagnostics as an AMS tool” (4.22 vs. 4.91, mean Δ0.70; *P*<0.0001), and “perception of AMS as an important topic” (4.96 vs. 5.00, mean Δ0.04; *P*=1.00). 

**Figure 2:**
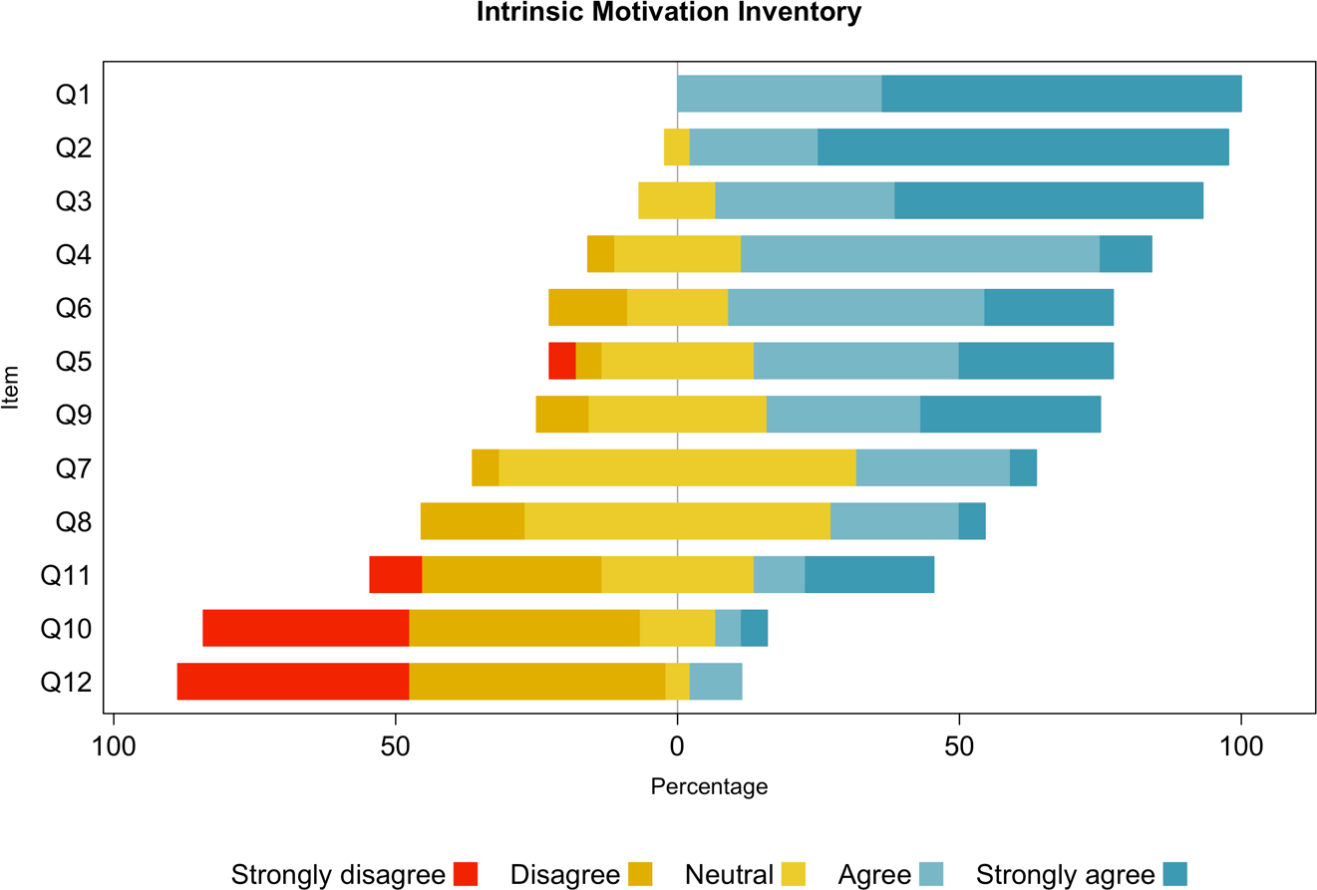
Distribution of participants’ (N=23) responses of the intrinsic motivation inventory on a Likert scale, per item

Regarding the intrinsic motivation inventory on the item level (see table 1 (Tab. 1) and figure 2 (Fig. 2)), we observed the highest scores for “I enjoyed doing the task very much” and “I would describe this activity as very interesting” (mean scores 4.6±0.5 and 4.7±0.6, each), followed by “doing the task was fun” (4.4±0.7), and then by “I am satisfied with my performance at this task”, “I believe I had some choice about doing this activity, and “In the course activity, I was able to proceed as I wished” (all 3.8±1.0). Neutral agreement was achieved for the items “I think I did pretty well at this activity” (3.3±0.6), “I think I am pretty good at this task” (3.1±0.8), and “I was concerned about whether I would be able to do the activity well in the course” (3.0±1.3). The two items with predominantly disagreeing statements were “I was anxious while working on this task” (2.0±1.0) and “I felt very tense while doing this activity” (1.8±0.9).

## Discussion

We show in the study herein the feasibility of an antimicrobial stewardship elective for medical undergraduate students, and the perceived increase in participants’ competencies. Of note, self-reported increase in competencies was observed across all domains and may be conclusively realistic (“if you teach them, they will learn”) or flawed [25]. In addition, the elective was perceived as joyful and very interesting by the majority of the participants. 

Knowledge gaps among medical students with regard to AMR and antibiotics are still pervasive [26], and there remains a need for standardization of possible interventions. A study from the UK by Castro-Sánchez and colleagues had shown that although the majority of UK universities reportedly included elements of antimicrobial stewardship in their curricula, the overall education on stewardship was deemed disparate and fragmentary [27].

We have previously shown in a sample of medical undergraduate students from German universities that despite the existence of the AMS objective in the NKLM, the vast majority of respondents reported to have had only scarce exposition to AMS-related teaching contents [28], highlighting the discrepancy between existing, formulated and documented objectives and the lack of learning experience. Wang and colleagues reported in a US study on the outcomes of an undergraduate elective course on AMS extending over a two-week period [19]. Similar to our intervention, they focused on self-assessment of the participants’ knowledge and comfort within AMS relevant core topics, for which they demonstrated a substantial increase from before to after the course for almost all items, albeit without obtaining an objective outcome parameter.

Thus, assessment is crucial as overconfidence effects [29], that have been detected earlier in other medical competencies like hand hygiene [30] and basic life support [31], limit self-assessment and inhibit metacognition. The discalibration effects [32] have not been evaluated at this point of the study due to a small sample size, but should be considered in the future for better understanding of self-assessment and competence in AMS, and the possibility of the existence of motivation patterns with different educational and motivation needs [33]. 

In a review of educational AMS contents in medical schools, Augie and colleagues found high heterogeneity in the type and extent of interventions, and also highlighted the need to assess long-term retention of acquired skills and knowledge [34].

There are some strengths of our study that deserve mentioning. First, we made use of an intrinsic motivation inventory tested in other educational settings generally affirming its use to be feasible. This will allow future studies for a deeper analysis of how much the new teaching format affected individual participants’ emotional state, for which there is hardly any comparable data in the published literature. In addition, the instrument’s reliability, criterion- and construct-validity in the context of AMR education leaves room for future investigations in larger and more representative populations. Second, we used a previously published set of self-assessment questions to obtain data on the knowledge gain. Third, our approach entailed a sustainable way of using available virtual case vignettes from the CASUS database. Fourth, the mixture of teaching methods employed in our intervention – case-based learning, problem-based learning, participation in ward rounds, etc. – may have been one reason, alongside the interprofessional and interdisciplinary team consisting of clinicians, microbiologists, pharmacists, infection preventionists, why the activities were rated by most participants as joyful and very interesting. The interdisciplinarity of our approach may be a major facilitator for longitudinal implementation across different subjects of the clinical study phase. 

Our study also has limitations. First, this is a single-center experience with a rather small sample size, which however complements data reported by other groups [19]. Second, the voluntary nature of the elective course introduced the possibility of selection bias, as only participants with a high a priori appreciation for the topic (in our case a sample with a strong gender spin) may have enrolled in the first place. Third, our data focus on self-assessment and intrinsic motivation, while we lack other outcome parameters, including and objective assessment of acquired competencies, and those relevant for long-term knowledge retention. Fourth, we did not assess the potential curriculum implementation, which will likely necessitate elements of blended-learning to balance the time needed for the small-group sessions. 

An aspect that has been understudied in the past remains an interdisciplinary, cross-sectoral approach to the issue of antimicrobial resistance [35]. Other, related disciplines have initiated endeavors similar to the one presented herein, e.g., the standardized curriculum for veterinary medicine students [36]. We believe – and we have made a case for this previously [28] – that these efforts should be aligned, taking a One-Health approach to appreciate that AMR necessitates a cross-sectoral approach as it affects not only the human sector, but also animals, plants, and the environment. Innovative educational tools may serve to facilitate the implementation and uptake of AMR and AMS related contents, such as game-based learning, gamification, or serious games, although extensive evaluations with an assessment of long-term outcomes are usually scarce [37].

## Conclusions

The findings of our study are of interest to policy makers and curriculum development teams, not only at German medical faculties. However, the long-term implementation of such a highly immersive teaching format will depend on enough time slots due to the small course sizes. One possible trade-off could be achieved by moving lecture-based contents into asynchronous lectures, which the students can (re-)watch at their discretion. Likewise, a higher number for instructors may be necessary for the actual case-based learning elements, for which also student instructors could be deployed [18]. Finally, since the objectives of the course address not only knowledge but also attitude, the ideal mode of student assessment may very well go beyond mere multiple-choice question formats. Future challenges of our format pertain to the implementation in the core curriculum, ideally in a longitudinal fashion while maintaining the interprofessional and interdisciplinary approach, and an assessment of short- and long-term gains in competencies.

## Notes

### Authors’ ORCIDs

- Cihan Papan: [0000-0001-7951-2567]
- Barbara C. Gärtner: [0000-0002-5234-7634]
- Arne Simon: [0000-0001-9558-3330]
- Rachel Müller: [0000-0003-0562-2901]
- Martin R. Fischer: [0000-0002-5299-5025]
- Dogus Darici: [0000-0002-2375-8792]
- Sören L Becker: [0000-0003-3634-8802]
- Katharina Last: [0000-0002-0538-1901]
- Stefan Bushuven: [0000-0001-6272-0714]

### Shared Authorship

The authors Katharina Last and Stefan Bushuven share the last authorship.

## Acknowledgements

We are grateful to Silke Mahler, Silke Müller, and Dominik Monz for secretarial and organizational support. Additionally, we would like to acknowledge support from Norbert Graf, Thomas Gilcher, Guy Danziger, Philipp M. Lepper, Carlos Metz, Matthias Schröder, and Frederic Albrecht.

## Competing interests

The authors declare that they have no competing interests. 

## Figures and Tables

**Table 1:**
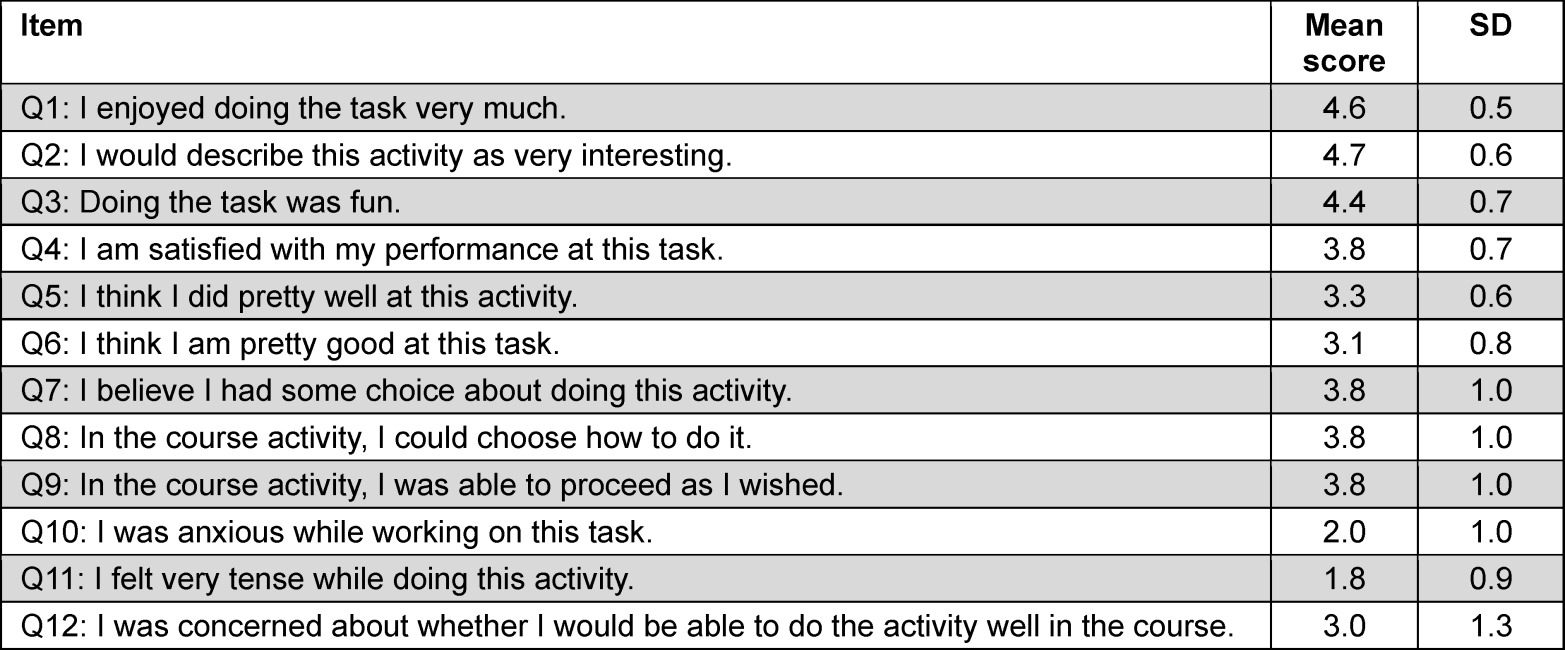
Mean scores and standard deviations (SD) of each item of the intrinsic motivation inventory
